# Characterization of Volatile Compounds and Odorants in Different Sichuan Pepper Varieties in Tallow Hotpot

**DOI:** 10.3390/foods14040627

**Published:** 2025-02-13

**Authors:** Wenhua Li, Qiaojun Wang, Huilin Huan, Gangcheng Wu, Qingzhe Jin, Youfeng Zhang, Xingguo Wang

**Affiliations:** 1 State Key Laboratory of Food Science and Resources, School of Food Science and Technology, Jiangnan University, Wuxi 214122, China; 6220112043@stu.jiangnan.edu.cn (W.L.); qiaoqiaosimidar@163.com (Q.W.); 6220111040@jiangnan.edu.cn (H.H.); gangcheng.wu@jiangnan.edu.cn (G.W.); jqzwuxi@163.com (Q.J.); 2 Department of Flavor Chemistry, Institute of Food Science and Biotechnology, University of Hohenheim, Fruwirthstr. 12, 70599 Stuttgart, Germany

**Keywords:** Sichuan pepper, Sichuan hotpot, volatile compounds, GC-MS, chemometrics analysis

## Abstract

Sichuan pepper plays a vital role in enhancing the flavor of hotpot. However, the specific flavor compounds involved are still unclear. In this study, the key aroma components of Sichuan pepper tallow hotpot were explored. Six aroma attributes were evaluated by quantitative descriptive sensory analysis (QDA). Gas chromatography–mass spectrometry (GC-MS) identified 56 compounds. Among them, a total of 27 aroma-active compounds were identified by gas chromatography–olfactometry (GC-O) and aroma extract dilution analysis (AEDA). Sixteen aroma-active compounds were determined using odor activity values (OAVs) ≥ 1. Linalool, linalyl acetate, *D*-limonene, sabinene, *β*-myrcene, eucalyptol, *α*-terpineol, terpinen-4-ol, acetic acid, (*E*,*E*)-2,4-decadienal, (*E*)-2-heptenal, and others were identified as the key aroma compounds. Chemometrics analysis indicated that the aroma of green Sichuan pepper tallow hotpot was green, and the aroma of different red Sichuan pepper tallow hotpots varied significantly. The research results serve as a foundation for the quality control and production of the hotpot industry.

## 1. Introduction

Sichuan pepper is a member of the *Zanthoxylum genus*, which is part of the Rutaceae family and includes over 250 species. This typically denotes *Zanthoxylum bungeanum* Maxim (*Z. bungeanum*, red Sichuan pepper) and *Zanthoxylum schinifolium* Sieb. et Zucc. (*Z. schinifolium*, green Sichuan pepper) [1,2]. It is also known as Huajiao, Chinese pepper, and Chinese prickly ash and is primarily grown in the southwest regions of China. The dried pericarp is commonly used as powder, Sichuan pepper salt, and Sichuan pepper oil, especially in Sichuan hotpot, a traditional Chinese dish known for its unique flavor [3]. Sichuan pepper provides a pleasant aroma (volatile compounds) and numbing sensation (non-volatile compounds, alkylamides), making it a key spice in the dish [4,5]. Apart from its culinary uses, Sichuan pepper also possesses bioactive properties such as antioxidant, anti-inflammatory, and antibacterial activities [6]. Sichuan hotpot is prepared by frying vegetable oil or animal oil, along with Sichuan peppers, chili peppers, bean paste, and other seasonings, at high temperatures [7]. Sichuan hotpot is the leading force in China’s catering industry, accounting for approximately one-third of the sector’s revenue in 2021. It is expected to reach 668.9 billion (approximately USD 92.3 billion) by 2025 [8,9]. Sichuan pepper tallow hotpot is made by stir-frying Sichuan pepper in high-temperature tallow. Due to the diverse seasonings and complex aroma composition of the Sichuan hotpot, it is crucial to explore the influence of the key ingredient, Sichuan pepper, on its flavor.

Volatile compounds play a crucial role in assessing quality, impacting consumer preferences and product competitiveness. The scent of the Sichuan pepper was influenced by a variety of factors, such as the varieties, climate conditions (sunlight, rainfall, temperature), and growing conditions [10]. Volatile substances such as linalool, linalyl acetate, *D*-limonene, and sabinene could be used to distinguish different varieties [11]. The main compounds in red Sichuan pepper were terpenes, while the main compounds in green Sichuan pepper were terpene alcohols [12]. Different geographical factors play a role in the volatile substances found in Sichuan pepper, as its growing regions and climate environments vary widely. In the southwest and northwest regions, the Sichuan pepper contains higher levels of limonene and linalool, while in the north, east, and central regions, the Sichuan pepper contains higher levels of *β*-myrcene and (*E*)-ocimene [10]. Therefore, it is necessary to explore the different varieties of Sichuan pepper in the aroma of tallow hotpot processing.

Volatile substances could be identified, but only key aroma-active compounds have a significant impact on the overall aroma of food. Previous studies mainly focused on analyzing the aroma of Sichuan pepper fried in vegetable oil [13]. *β*-Myrcene, (*E*)-2-heptenal, limonene, *α*-terpineol, and *p*-cymene were identified as the main aroma-active compounds of five fried peppers in rapeseed oil. Linalool, linalyl acetate, and 1,8-cineole were key aroma substances used to differentiate red and green Sichuan pepper oil [14]. Likewise, (*E*)-2-heptenal, 1,8-cineole, *β*-myrcene, *β*-ocimene, limonene, and linalool were primary key aroma compounds in fried Hanyuan and Hancheng pepper in rapeseed oil. *β*-Phellandrene, *p*-cymene, acetic acid octyl ester, octanal, citronellol, and sabinene could distinguish the Hanyuan and Hancheng pepper oil [15]. There is only one reported study on the aroma compounds of Sichuan pepper fried in tallow. Linalool, linalyl acetate, terpinen-4-ol, *D*-limonene, *α*-terpinyl acetate, and others were identified as contributing significantly to the overall aroma profile [16]. According to the literature summary and analysis of Sichuan pepper pericarp oil, raw Sichuan pepper, and stir-fried pepper tallow’s main aroma compounds (Table S1), they primarily consist of terpenes, alcohols, esters, aldehydes, and other substances [12,16,17]. Linalool, *γ*-terpinene, and *D*-limonene were common substances found in all three samples. Raw Sichuan pepper and Sichuan pepper pericarp oil both contain many terpenes, but Sichuan pepper pericarp oil’s aromatic concentration is notably low. Stir-fried pepper tallow contains more alcohols, esters, and aldehydes, which may be due to the frying process promoting the release of raw pepper’s aromatic substances and generating more aromatic compounds, thereby increasing consumer preference. Further exploration is needed to analyze the key aroma compounds of different commonly used Sichuan pepper varieties in the hotpot process.

Therefore, this study aimed to (a) classify the different aromas of Sichuan pepper tallow hotpot by quantitative descriptive sensory analysis (QDA); (b) detect volatile compounds in samples by GC-MS; (c) identify key aroma-active compounds and assess aroma contribution using aroma extract dilution analysis (AEDA) and odor activity value (OAV); and (d) analyze the correlations and difference of Sichuan pepper tallow hotpot, characteristic aroma compounds, and sensory attributes by multiple chemometric methods. The results of this study could provide a data basis to improve the flavor of Sichuan hotpot.

## 2. Materials and Methods

### 2.1. Materials and Chemicals

These Sichuan pepper varieties were purchased from the local wholesale market in Chengdu. The Sichuan pepper varieties are named after the place name and the color of the peel: Jiangjin green Sichuan pepper (JJGSP), Jinyang green Sichuan pepper (JYGSP), Hanyuan green Sichuan pepper (HYGSP), Hancheng red Sichuan pepper (HCRSP), Hanyuan red Sichuan pepper (HYRSP), and Wudu red Sichuan pepper (WDRSP), each with a moisture content of less than 11%. Beef tallow (BT) was acquired from Guanghan Maidele Food Co., Ltd. (Chengdu, China).

Linalool (≥98%), linalyl acetate (≥96%), eucalyptol (≥99%), terpinen-4-ol (≥98%), *β*-myrcene (≥90%), sabinene (≥98%), (*E*,*E*)-2,4-decadienal (≥90%), acetic acid (≥99%), *β*-caryophyllene (≥90%), *α*-terpineol (≥98%), *(E)*-*β*-ocimene (≥95%), *(E)*-2-decenal (≥95%), and *α*-terpinyl acetate (≥85%) were purchased from Yuanye Biotechnology (Shanghai, China). *D*-limonene (≥95%), *β*-phellandrene (≥95%), and (*E*)-2-heptenal (≥95%), along with standard alkanes (C_8_–C_40_), were purchased from Sigma-Aldrich (Shanghai, China). High-performance liquid chromatography (HPLC)-grade methanol and the internal standard 1,2-dichlorobenzene (99%) were obtained from Aladdin Industrial Corporation (Shanghai, China).

### 2.2. Preparation of Sichuan Hotpot

One hundred grams of deseeded Sichuan pepper were taken and then rinsed to remove surface dust with water. The excess moisture was then drained before setting it aside. Five kilograms of refined BT were put into a commercial cooking hot plate (Manchu, China) and heated to a temperature of 110 °C. The Sichuan pepper was added to the heated BT and stirred, keeping it for 5 min. The mixture was allowed to cool naturally to 60 °C, and then the Sichuan pepper was filtered out, yielding the hotpot sample. The final product was then packaged in airtight bags and stored at a temperature of −18 °C for preservation. BT heated under the same conditions as the experimental setup, but without the addition of Sichuan pepper, served as the control in the experiment.

### 2.3. Sensory Analysis

A sensory assessment panel comprising 12 members (an equal gender mix of women and men, aged 20 to 30, and all from Jiangnan University) with profound expertise in hotpot sensory assessment conducted a quantitative descriptive sensory analysis (QDA) at ambient temperature, with some adjustments to the analysis method based on the existing literature [15]. Members are trained according to GB/T 16291 [18,19]. The panel began by enumerating all flavor attributes associated with Sichuan hotpot, followed by a collaborative deliberation to identify key sensory characteristics: green, floral, pine, citrus-like, fatty, and cowy. The intensity of each sensory attribute was rated on a scale that increased from 1 (not noticeable) to 10 (very strong), with each unit representing a small increase. Each 5 g sample was precisely measured into a 100 mL sniff bottle with screw tops at room temperature 25 °C. The samples were randomly given three-digit codes, and each one was assessed three times by the panelists, with a 5 min break between evaluations. The final aroma profile score was averaged from three parallel experiments.

### 2.4. Headspace Solid-Phase Microextraction

According to the method with some adjustments, after conducting preliminary experiments, the headspace solid-phase microextraction (HS-SPME) extraction fiber with a 50/30 μm DVB/CAR/PDMS (Supelco, Bellefonte, PA, USA) coating was chosen for the extraction process [14]. The fiber was installed into a TriPlus RSH automatic sampler (PAL RSI 85, CTC Analytics AG, Zwingen, Switzerland). The fiber was initially conditioned at 250 °C for 3 min and then aged at the same temperature for 2 min before each extraction. Five grams of the sample and 1 μL of the internal standard (1,2-dichlorobenzene, 1.306 μg/mL in methanol) were mixed in a 20 mL headspace vial. The vial was placed in a temperature-controlled water bath at 60 °C with a magnetic stirrer set at 100 rpm for 25 min to reach equilibrium, followed by an extraction time of 30 min. After extraction, the fiber was taken out and inserted into the GC-MS injection port for thermal desorption at 250 °C for 5 min.

### 2.5. GC-MS Analysis

The aroma compounds in each hotpot sample were separated and identified through GC-MS (7890B GC System, 5977B MSD, Agilent Technologies, Santa Clara, CA, USA) with DB-WAX column (30 m × 0.25 mm × 0.25 μm, Agilent Technologies, USA), according to the method of the literature with some modifications [14]. Helium was used as the carrier gas with a purity level of ≥99.99%. The pressure was maintained at 47.7 kPa and a flow rate of 1.0 mL/min. The initial temperature of the oven was maintained at 40 °C for 1 min, then ramped linearly to 80 °C at a rate of 4 °C/min, followed by increments to 120 °C at 4 °C/min, and finally increased to 210 °C at 6 °C/min, where it stayed for 5 min. The electron impact ionization source has an ionization energy of 70 eV, as well as an interface temperature of 250 °C. A solvent delay of 3 min was applied, with the mass scan range from 50 *m*/*z* to 550 *m*/*z* in full scan mode.

### 2.6. GC-O and AEDA

An Agilent 7890B GC (Agilent Technologies, USA) combined with a sniffing detector port (C200 ODP, Gerstel, Mulheim an der Ruhr, Germany) was used for the AEDA. Other GC parameters are the same as those in the GC-MS analysis. AEDA is the dilution of aroma-active compounds extracted by solvent-assisted flavor evaporation by increasing the solvent volume and shortening the extraction time. It can also be achieved by changing the inlet split ratio for aroma-active compounds extracted by SPME. Flavor dilution (FD) is the maximum dilution at which aroma-active flavor compounds can be smelled. Volatile compounds were extracted using HS-SPME, and the GC inlet split ratio was increased from 1:2 to 1:4, 1:8, 1:32, 1:64, and 1:128 for sniffing the sample odor, with three parallel experiments conducted for each sample, according to the method of the literature with some modifications [16].

### 2.7. Qualitative and Quantitative Analysis of Volatile Compounds

During this experimental procedure, volatile substances were characterized by analyzing their mass spectrometry spectral data with the 2020 NIST 20 MS database reference library. Concurrently, the retention times for a series of *n*-alkanes (C_8_–C_40_) were determined using the identical temperature ramping protocol, which allowed for the calculation of the retention index (RI) for the volatile compounds [15]. These indices were subsequently compared to values documented in the pertinent literature and those available on the NIST Chemistry WebBook. The substance was identified as the target substance when the *RI* value of the sample was less than 50 different from the theoretical *RI* value.

$$RI=100\times n+100\times \frac{t_{\left(x\right)}-t_{\left(Cn+1\right)}}{t_{\left(Cn+1\right)}-t_{\left(Cn\right)}}$$

The *RI* is calculated as follows: *t*(*x*), *t*(*Cn*), and *t*(*Cn* + 1) represent the retention times of compound *x*, normal alkane with *n* carbon atoms, and normal alkane with *n* + 1 carbon atoms, respectively.

A 5-point standard curve for aroma compounds was established using the external standard method. 1,2-Dichlorobenzene was blended with varying concentrations of an external standard solution. The resulting mixture was then combined with an odorless matrix created through molecular distillation. One hundred grams of beef tallow was fed into a molecular distillation apparatus under a vacuum of 10^−4^ kPa using the reference method. The feed rate was adjusted to 1.5 mL/min, and the temperatures of the feed tank, condenser, and evaporator were set to 80 °C, 40 °C, and 150 °C, respectively [20]. A standard curve was generated by plotting the peak area ratio of aroma compounds to the internal standard (y-axis) against the concentration ratio (x-axis), enabling precise quantification of aroma-active compounds.

### 2.8. Odor Activity Values

The calculation method of odor activity value (OAV) is to divide the concentration of aroma compounds (Ci) by the odor threshold detected in oil (OTi) (OAV = Ci/OTi), where OAV ≥ 1 indicates compounds that contribute to the overall aroma. All odor thresholds of the corresponding odorants in oil were from information available in the literature [14,16,21].

### 2.9. Statistical Analysis

The data from GC-MS were processed using SPSS Statistics 23.0. Sensory radar charts were created using Origin 2021. The histograms of relative content of volatile substances were plotted using GraphPad Prism 8.0.1. Partial least squares regression (PLSR) analysis was conducted using XLSTAT 2021. Principal component analysis (PCA), hierarchical cluster analysis (HCA), and orthogonal partial least squares–discriminant analysis (OPLS-DA) were performed using SIMCA 14.1. Variable importance in projection (VIP) was performed using MetaboAnalyst 6.0 (www.metaboanalyst.ca). A heat map was generated using TBtools 2.136.

## 3. Result and Discussion

### 3.1. Aroma Profiles of Sichuan Hotpot by QDA

**Figure 1:**
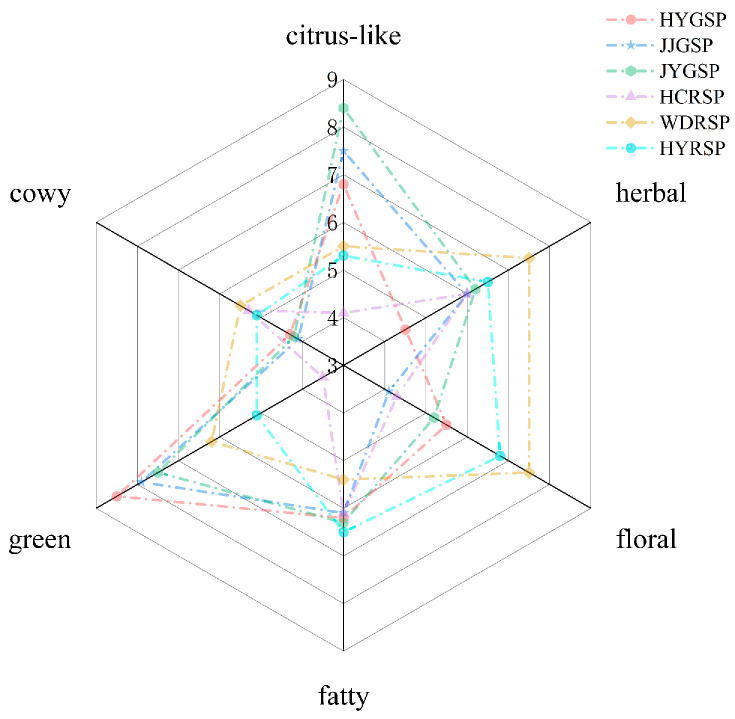
Radar map for sensory evaluation of flavor of Sichuan pepper tallow hotpot.

The aroma profiles of six Sichuan pepper tallow hotpot varieties were evaluated using QDA. The evaluation panel members chose six aroma attributes for analysis, including green, floral, herbal, citrus-like, cowy, and fatty. The radar chart illustrated the aroma profiles of various Sichuan pepper tallow hotpots, effectively presenting sensory data and showcasing flavor characteristics in a clear manner. This visualization aids in comparing different sample aroma types, as demonstrated in Figure 1. The WURSP and HYRSP tallow hotpot exuded a rich floral and herbal aroma, whereas the JYGSP tallow hotpot had a distinct citrus-like aroma. In contrast, the JJGSP tallow hotpot had a prominent green scent. The overall aroma of the HCRSP hotpot tallow was low, especially the green aroma. Compared to green, floral, herbal, and citrus-like notes, the cowy and fatty aroma in Sichuan tallow hotpots received a lower score. This might be due to the fact that the fragrance substances and content provided by Sichuan pepper in hotpot were greater than those in tallow, with minimal differences between samples. The tallow hotpots exhibited different aroma characteristics, likely influenced by the types and amounts of volatile compounds present in different Sichuan pepper varieties. From the color of Sichuan pepper, the tallow hotpot with green Sichuan pepper exhibited predominantly green and citrus-like, while red Sichuan pepper displayed unique floral and herbal notes. Research has shown that limonene, *β*-phellandrene, and myrcene are the substances that give Sichuan pepper its strong green and pine leaf notes [6]. Likewise, fried different types of Sichuan pepper in rapeseed oil made red Sichuan pepper oil with herbal, spicy, fatty, and pine-like characteristics. Conversely, green Sichuan pepper oil had a green and citrus-like aroma [14].

### 3.2. Volatile Compounds Analysis of Different Sichuan Pepper Tallow Hotpots by SPME-GC-MS

**Figure 2:**
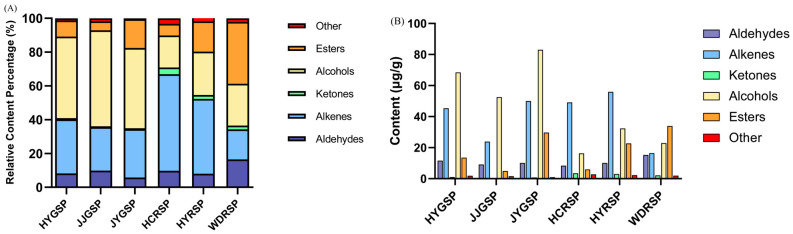
Differences of volatile compounds in six Sichuan pepper tallow hotpots. (**A**) The relative content percentage of volatile compounds categories. (**B**) The average relative volatile compounds content in each category.

Volatile compounds were extracted with HS-SPME and analyzed by GC-MS. The composition of volatile compounds differed among the six types of Sichuan pepper tallow hotpot, which could account for the variations in aroma profiles (Figure 2). A total of 56 volatile compounds were identified, including 20 aldehydes, 15 alkenes, 10 alcohols, four ketones, four esters, and three others. The WDRSP and HYRSP samples contained the most volatile compounds (46 and 44), while the JYGSP sample had the fewest volatile compounds (35).

In the JJGSP, HYGSP, and JYGSP tallow hotpots, alcohols were the most abundant (56.74%, 48.21%, and 47.55%, respectively), followed by alkenes (25.75%, 31.79%, and 28.66%) and esters (5.35%, 9.54%, and 16.99%). Conversely, in the HCRSP and HYRSP samples, alkenes had the highest abundance (57.14% and 44.27%), followed by alcohols (18.92% and 25.62%) and esters (6.9% and 17.96%). In the WDRSP sample, esters (36.61%) were the most abundant, followed by alcohols (24.73%) and alkenes (17.74%). Different aroma types could have been shaped by the content and combination of various classes of volatile compounds. Over half of the total volatile compounds in Sichuan pepper tallow hotpot were composed of alcohols and alkenes, leading to the presence of green, floral, herbal, and citrus-like notes. Additionally, these compounds could be influenced by factors such as geography, climate, ripeness, and temperature [22]. Research found that the total volatile substances in Sichuan pepper ranged from 1976.64 to 2353.55 mg/kg at different growth stages, with the highest content seen during the slight red stage of the fruit peel. Terpene content was at its lowest (50.01%) during the light red stage, with alcohols increasing as Sichuan pepper fruits matured, reaching a peak (28.60%) at the red stage [23]. The content of *α*-caryophyllene, allo-ocimene, and phellandrenhydrat increases with lower temperatures and precipitation, as well as higher wind speeds [10]. Research has found that red Sichuan pepper exhibited a notably higher total ester content than green Sichuan pepper, while green Sichuan pepper showed a significantly higher total alcohol content than red Sichuan pepper [11]. Volatile compounds were found to be released during the frying of Sichuan pepper in tallow hotpots. The JYGSP, HYGSP, and JJGSP tallow hotpots also showed a high alcohol content. Therefore, tallow hotpot fried with different varieties of Sichuan pepper exhibits distinctly different aromas.

### 3.3. Aroma-Active Compounds in Six Sichuan Pepper Tallow Hotpots

The AEDA was utilized to analyze the odorants that contribute to the overall aroma. Twenty-seven aroma-active compounds were detected by GC-MS analysis and AEDA in the Sichuan pepper tallow hotpots, among which 21, 22, 23, 24, 25, and 25 aroma-active compounds were identified in HYGSP, JJGSP, JYGSP, HCRSP, HYRSP, and WDRSP, respectively (Table 1). The higher the FD factor of a compound, the stronger its impact on the overall aroma of the samples [24]. Linalool (spicy, citrus-like, green, floral), linalyl acetate (woody, herbal), *D*-limonene (citrus-like), and *β*-myrcene (spicy, balsamic, rosin), with a high FD factor (128), indicating that these aroma compounds may have a high contribution to the aroma of Sichuan pepper tallow hotpot. The linalool in the JYGSP, JJGSP, and HCGSP tallow hotpot had an FD factor of 128, while in the HYRSP, WDRSP, and HCRSP tallow hotpot, the FD factor for linalool was 64, 64, and 8 respectively. In the HCRSP sample, only *D*-limonene had an FD factor of 128. Therefore, there were some differences in the final overall aroma presented by different samples. Sabinene (woody, citrus-like), eucalyptol (herbal), *α*-terpineol (pine-like, woody), *β*-phellandrene (minty), and terpinen-4-ol (spicy, musty) demonstrated FD factors (16), indicating their positive contribution to the aroma characteristics of Sichuan pepper tallow hotpot.

The FD factor values of the same aroma-active substance may vary in different samples, possibly due to the use of various Sichuan pepper varieties in tallow hotpot. It was discovered through research that linalool, linalyl acetate, *D*-limonene, and sabinene were key odorants that differentiate between red and green Sichuan pepper [11]. The FD values of aroma compounds usually correlate positively with the overall aroma contribution of the sample, with FD factors ≥ 8 identified as potential key aroma compounds, resulting in the screening of 15 compounds [25]. Previous studies reported linalool, linalyl acetate, *D*-limonene, and other compounds as important aroma compounds [14,16]. These aroma compounds were crucial for the overall citrus-like, woody, floral, herbal, and green notes of Sichuan pepper tallow hotpot.

However, the aroma compounds detected using GC-O and AEDA may vary considerably when they are in the air as a matrix as opposed to when they are in the actual sample matrix of tallow. This difference could lead to varying odor threshold values. Therefore, we also calculated the OAV to better understand the contribution of aroma-active compounds to Sichuan pepper tallow hotpots.

### 3.4. Quantitation of Key Aroma-Active Compounds and OAV Analysis

A total of fifteen aroma-active compounds (FD factors ≥ 8) were measured in Sichuan pepper tallow hotpots using standard reference materials (Table 2). In the HYGSP tallow hotpot, the highest levels were found for *D*-limonene (209.9 ± 10.52 μg/g) and linalool (200.27 ± 2.73 μg/g), followed by linalool, *β*-myrcene, and sabinene. Similar results were seen in the JJGSP and JYGSP tallow hotpots, where *D*-limonene (141.64 ± 1.93 μg/g, 268.84 ± 5.58 μg/g) and linalool (148.58 ± 3.63 μg/g, 240.74 ± 13.47 μg/g) were the most abundant compounds. Linalyl acetate (121.05 ± 10.34 μg/g, 136.91 ± 1.76 μg/g) was highest in the JYGSP and WDRSP tallow hotpots. Notably, *D*-limonene (350.67 ± 16.08 μg/g) was the most prominent in the HYRSP tallow hotpot, showing significant differences in content compared to linalool, linalyl acetate, and *β*-myrcene. These compounds are considered to be the key aroma components in Sichuan pepper, with *D*-limonene playing a significant role in its distinctive citrus-like scent [26].

The aroma contribution of aroma-active compounds to Sichuan pepper tallow hotpots could be gauged through OAV, which is influenced by both their concentration and odor threshold in the given matrix [27]. Compounds with an OAV ≥ 1 were identified as potential contributors to the sample, with higher values indicating a greater contribution.

Linalool in tallow had an odor threshold of 1.08 μg/g, significantly higher than the odor threshold in rapeseed oil (0.037 μg/g) [16]. The OAV of linalool in the JYGSP tallow hotpot (240.74 ± 13.47 μg/g) was higher (222), followed by the HYGSP and JJGSP, while the OAV of linalool in the HCRSP tallow hotpot (10.66 ± 0.74 μg/g) was the lowest at 9. Similarly, (*E*)-2-heptenal had a low overall concentration; however, with a threshold as low as 0.001 μg/g, it resulted in a high OAV, ranging from 1163 to 2015, in hotpot samples. Green Sichuan pepper tallow hotpot has a higher linalool content than red Sichuan pepper tallow hotpot due to differences in the varieties of Sichuan pepper. Linalool gives off a floral scent when present in low concentrations, but at higher concentrations, it contributes to a spicy aroma [28]. Linalyl acetate has the highest OAV in the WDRSP tallow hotpot (OAV = 121), followed by the JYGSP tallow hotpot (OAV = 107), and the lowest in the HCRSP tallow hotpot (OAV = 6). The precursor substance of *α*-terpineol was found to be linalool in research studies [29]. *D*-limonene in the HYRSP tallow hotpot (350.67±16.08 μg/g) had the highest content among all aroma-active compounds, but with an OAV of 167, its contribution was not the highest, which was related to the corresponding threshold in tallow (2.11 μg/g). (*E*)-*β*-Ocimene, with a sweet and herbal aroma, was found in small amounts (FD factor = 32, 6.01 ± 0.6 μg/g) in the HCRSP tallow hotpot. Despite its low concentration, it has the highest OAV of 177 due to its low threshold of 0.034 μg/g. *β*-Myrcene was higher in the HCRSP tallow hotpot (OAV = 128) and HYRSP tallow hotpot (OAV = 107) compared to other samples. The concentration and OAV value of eucalyptol are both relatively low. Eucalyptol, with its minty, sweet, and herbal notes, was detected in the HCRSP, HYRSP, and WDRSP tallow hotpots, with an OAV of 16, 8, and 3, respectively. However, the compound was not found in green Sichuan tallow hotpots, potentially leading to a less intense herbal aroma in those samples.

Linalool, linalyl acetate, and *D*-limonene exhibited high OAVs in tallow hotpots, which greatly influenced the overall aroma [21,30]. Research found that the most abundant aroma-active compound in hotpot seasoning was linalool (88.76 mg/kg), with an OAV of 42, contributing the highest aroma. Linalyl acetate and *D*-limonene were also found in high concentrations both before and after boiling the hotpot and were later confirmed to be key aroma-active compounds contributing to the overall aroma of the hotpot [7]. In Sichuan pepper tallow hotpots, these compounds, along with *β*-myrcene, were recognized as key aroma-active compounds with high OAVs and FD factors, thus playing a significant role in shaping their aroma.

According to Table S2, the volatile substances in beef tallow were mainly aldehydes, with nonanal (34.11%), octanal (19.87%), and hexanal (18.16%) having the highest relative content. (*E*)-2-Heptenal, (*E*,*E*)-2,4-decadienal, and (*E*)-2-decenal imparts a distinct fatty and cowy flavor to tallow hotpot, possibly arising from the processing of tallow, especially (*E*)-2-heptenal had the highest OAV. The major source of (*E*,*E*)-2,4-decadienal was linoleic acid [31]. Studies showed that aldehydes were primarily formed through the oxidation of lipids, with six-carbon unsaturated aldehydes being common products that further oxidize into shorter-chain aldehydes. Aldehydes with three to four carbons tend to have a sharp taste, while those with five to nine carbons impart a fresh, buttery flavor. Additionally, some higher molecular weight aldehydes could contribute to a citrus-like aroma [30].

### 3.5. PCA and OPLS-DA

**Figure 3:**
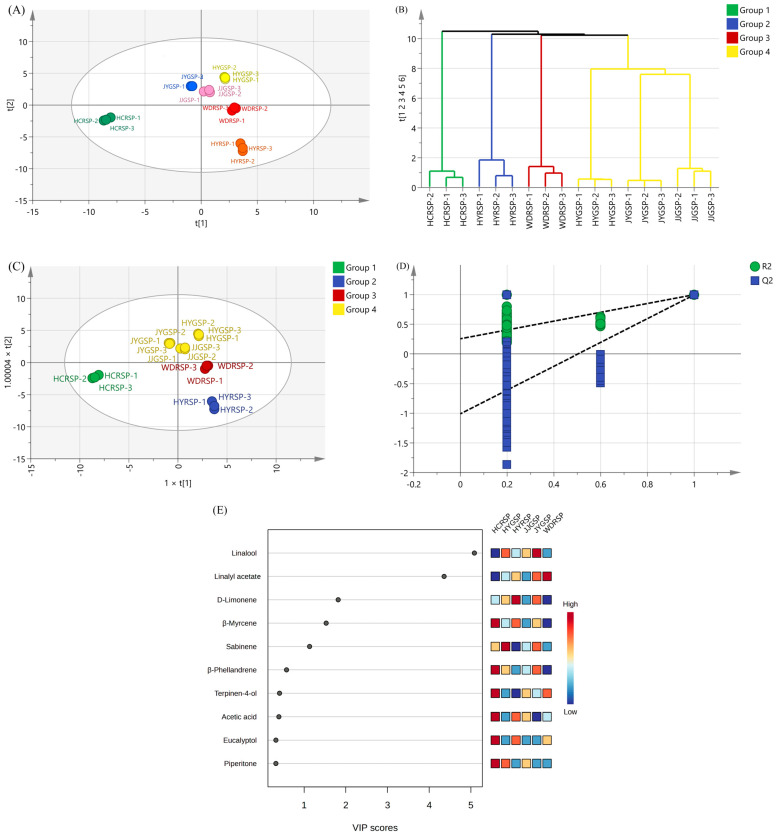
PCA and OPLS-DA of volatile compounds from six Sichuan pepper tallow hotpots. (**A**) PCA. (**B**) HCA. (**C**) OPLS-DA. (**D**) Distribution plot. (**E**) VIP analysis.

The volatile compounds of Sichuan pepper tallow hotpots were characterized using PCA and HCA (Figure 3A,B). PCA is a technique used in multivariate statistical analysis [32]. The clustering of points in the plot indicated high similarity among some samples, while more dispersed points showed lower similarity. The analysis showed that the variety of Sichuan pepper tallow hotpots had a significant impact on the flavor, R^2^ = 0.987, Q^2^ = 0.918, demonstrating that the Sichuan pepper varieties could influence the flavor profile of tallow hotpots. The samples were divided into four groups by HCA: group 1 included HCRSP, group 2 included HYRSP, group 3 included WDRSP, and group 4 included HYGSP, JJGSP, and JYGSP. The differences in volatile compounds among the hotpot samples were found to be significant.

OPLS-DA is superior to principal component analysis in elucidating differences between samples, as it can perform regression analysis on multiple independent variables to multiple dependent variables, identifying key variables affecting the samples more accurately [33]. Therefore, the model was chosen for further investigation into the disparities of aroma compounds in hotpots. Based on Figure 3C, the cumulative statistics of the fitted model were R^2^X = 0.986, R^2^Y = 0.998, and Q2 = 0.994, which were close to 1, indicating that the OPLS-DA model has good predictive ability for analyzing aroma compounds in hotpots. The distances between HYGSP, JJGSP, and JYGSP tallow hotpots were close, showing high similarity. There was a notable contrast observed between the HCRSP and HYRSP samples, mirroring the PCA results. The permutation test (*n* = 200) confirmed the reliability of the model, with R^2^ = 0.225 and Q^2^ = −1.01, indicating a reliable model with a low risk of overfitting.

The variable importance of projection (VIP) components could demonstrate the contribution of aroma compounds to the classification of the model. The higher the VIP, the greater the difference in aroma components between groups, making it more important for discriminating aroma types [34]. According to Figure 3D, linalool, linalyl acetate, *D*-limonene, *β*-myrcene, and sabinene had VIP > 1, indicating significant differences. As mentioned previously, the OAV and FD aroma contribution of these five compounds differ significantly between samples and thus could be considered crucial aroma compounds for distinguishing various Sichuan pepper tallow hotpots.

### 3.6. Heat Map Analysis

**Figure 4:**
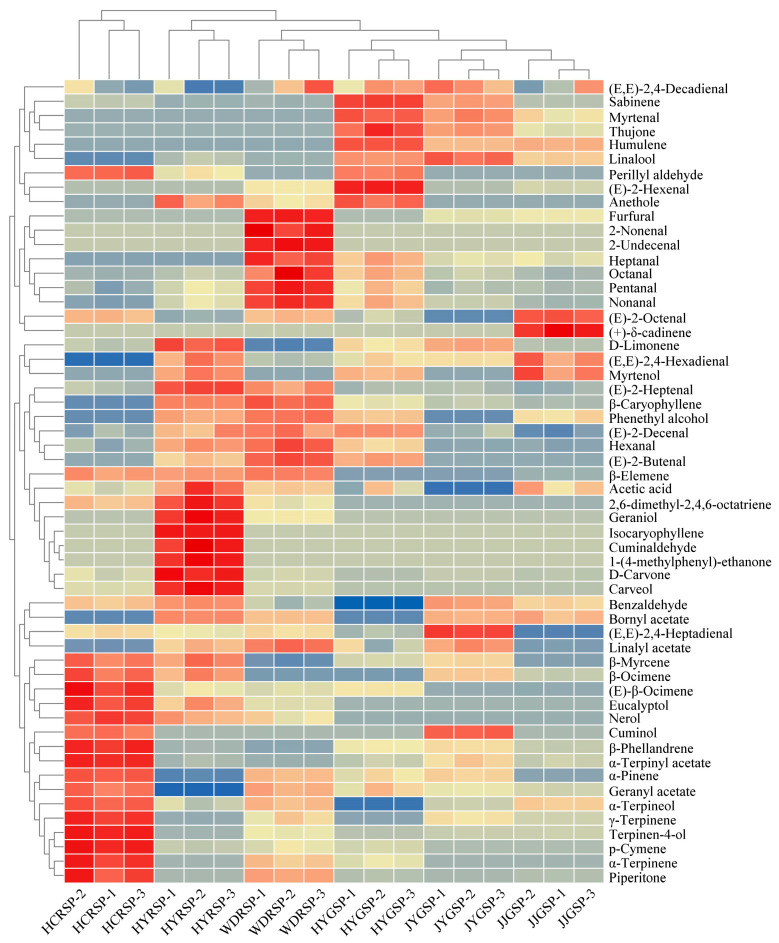
Volatile compounds in six Sichuan pepper tallow hotpots of heat map analysis.

To make a clearer comparison among six Sichuan pepper hotpot volatile compounds, a heat map will be utilized (Figure 4). The heat map in the figure indicated the levels of volatile substances in each sample using colors based on HCA. The colors range from blue (representing the minimum value of −2) to red (representing the maximum value of 2), showing the content of volatile components from high to low [35]. This result revealed that the samples were presented independently in six groups. From the heat map, it could be seen that the compounds with higher content in the HCRSP tallow hotpot were *α*-pinene, *β*-myrcene, *γ*-terpinene, terpinen-4-ol, and *β*-ocimene. In the HYRSP, the compounds with higher content were carveol, D-carvone, cuminaldehyde, *D*-limonene, geraniol, and (*E*)-2-heptenal. The WDRSP tallow hotpot had a higher content of nonanal, heptanal, octanal, 2-undecenal, and furfural. The HYGSP tallow hotpot contained higher levels of (*E*)-2-heptenal, perillyl aldehyde, linalool, humulene, and sabinene. The JYGSP tallow hotpot had a higher content of linalool, (*E*,*E*)-2,4-heptadienal, humulene, sabinene, and (*E*,*E*)-2,4-decadienal. The JJGSP tallow hotpot contained higher levels of (*E*)-2-octenal, (+)-*δ*-cadinene, and (*E*,*E*)-2,4-hexadienal.

HYGSP, JJGSP, and JYGSP tallow hotpot were characterized by high levels of alcohol compounds such as linalool (200.27 ± 2.73 μg/g, 148.58 ± 3.63 μg/g, 240.74 ± 13.47 μg/g), whereas HYRSP, WDRSP, and JYGSP tallow hotpot were abundant in ester compounds such as linalyl acetate (86.15 ± 0.42 μg/g, 136.91 ± 1.76 μg/g, 121.05 ± 10.34 μg/g). These trends aligned with the terpenoid composition of raw Sichuan peppers: red peppers showed higher levels of linalyl acetate and *D*-limonene, while green peppers were richer in linalool and *D*-limonene [11]. These substances fell under the category of terpene compounds. Research reports indicated that different raw Sichuan pepper varieties had high terpene content, accounting for half of the total volatile substance content (67.12%) [36]. Terpenes, terpene alcohols, and terpene esters were identified as the main terpene compounds in raw red and green Sichuan pepper. The main compounds in red Sichuan pepper were terpenes, while the main compounds in green Sichuan pepper were terpene alcohols [12]. The difference in composition may lead to distinct aromatic characteristics in Sichuan pepper hotpot.

Terpene compounds are primarily produced through the 2-C-methyl-d-erythritol-4-phosphate (MEP) pathway operating in the plastids and the mevalonate (MVA) pathway occurring in the cytosol [37]. Terpene compounds in Zanthoxylum are mainly monoterpenes, primarily synthesized by the MEP pathway [38]. For example, terpene synthases can convert geranyl diphosphate into *β*-myrcene and *D*-limonene through a shared isomerization–cyclization reaction sequence [39].

### 3.7. Relationship Between Sensory Descriptors and Volatile Compounds

**Figure 5:**
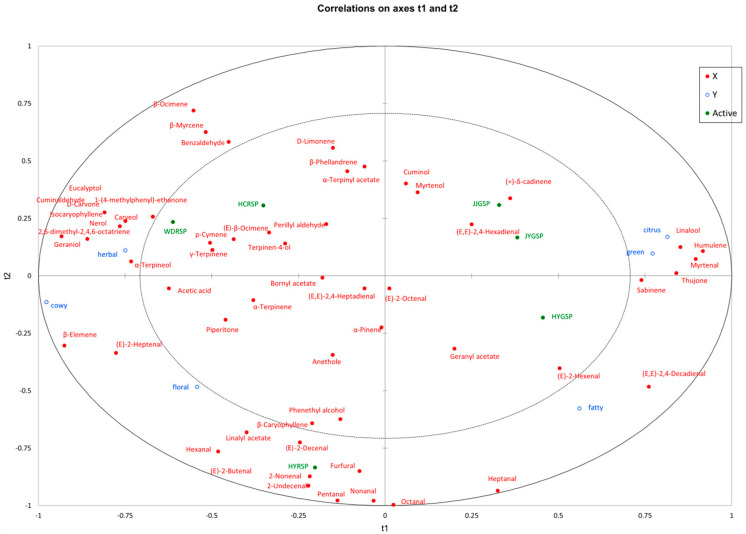
Correlation between sensory descriptors and volatile compounds by PLSR.

The flavor of samples could be influenced by the complex interactions between aroma perception and volatile compounds. In order to understand how six aroma sensory attributes and 56 volatile compounds affect flavor, a study was conducted using PLSR analysis (Figure 5). The model selected two factors based on the predicted sum of squared residuals, with the volatile component proportion of X variables at 82% and the sensory attribute proportion of Y variables at 52%. The inner ellipse represents 50% variance, and the outer ellipse represents 100% explained variance [40]. Generally, the data variables positioned between these two ellipses are well explained by the model. The considerable gaps between WDRSP, HCRSP, HYRSP, and HYGSP implied notable distinctions among these four samples of Sichuan pepper tallow hotpot. Conversely, the proximity of JJGSP and JYGSP suggested minimal variations between these two types of Sichuan pepper tallow hotpot. JJGSP and JYGSP exhibited the strongest correlation with the sensory attributes of citrus-like and green aroma, indicating that these two attributes are the prominent aroma features of JJGSP and JYGSP, aligning with the sensory analysis results depicted in Figure 1. Specifically, linalool, humulene, and thujone were closely associated with the sensory characteristics of citrus-like and green aromas. Linalyl acetate showed a strong association with floral scents, with higher concentrations in the HYRSP tallow hotpots. (*E*,*E*)-2,4-Decadienal was positively correlated with fatty aromas. *α*-Terpineol, eucalyptol, and *β*-myrcene were closely related to the sensory properties of herbal, and these compounds were the main aroma compounds in WDRSP and HCRSP tallow hotpots. Therefore, different Sichuan pepper tallow hotpots could be distinguished by their volatile substances and aroma attributes.

## 4. Conclusions

This study analyzed the aroma compounds in six Sichuan pepper tallow hotpots. Tallow hotpot exhibited six aroma attributes, according to QDA. JJGSP, JYGSP, and HYGSP were predominantly green and citrus-like, while HCRSP, WURSP, and HCRSP showed distinct floral and herbal notes. A total of 56 volatile compounds were detected, with 27 identified as aroma-active substances. Linalool, linalyl acetate, *D*-limonene, sabinene, *β*-myrcene, eucalyptol, *α*-terpineol, terpinen-4-ol, acetic acid, (*E*,*E*)-2,4-decadienal, (*E*)-2-heptenal, and others were identified as the key aroma compounds. The Sichuan pepper tallow hotpots were divided into four groups by chemometrics analysis. Additionally, the correlation between volatile compounds and aroma characteristics was examined using PLSR, showing that the green Sichuan pepper tallow hotpot displayed prominent citrus-like and green notes. Future research will concentrate on tracking the origins and changes in the dynamics of important aroma compounds during hotpot preparation in order to enhance flavor preservation and product quality.

## Figures and Tables

**Table 1:** The aroma-active compounds in six varieties of Sichuan pepper tallow hotpot were identified by AEDA.

No ^a^	Compounds	RI-(DB-WAX) ^b^	Theoretical RI ^c^	Odorant Description ^d^	FD ^e^						Identification Method ^f^
					HYGSP	JJGSP	JYGSP	HCRSP	HYRSP	WDRSP	
1	*α*-Pinene	1020	1015	Herbal, pine-like	2	2	2	2	2	2	RI/MS/O
2	Sabinene	1116	1113	Woody, citrus-like	32	8	16	8	8	8	RI/MS/O/S
3	*β*-Myrcene	1170	1165	Spicy, balsam	64	32	64	128	128	32	RI/MS/O/S
4	*D*-Limonene	1189	1185	Citrus-like	128	64	128	32	128	64	RI/MS/O/S
5	*β*-Phellandrene	1195	1212	Minty	64	32	64	64	32	8	RI/MS/O/S
6	Eucalyptol	1215	1212	Herbal	2	-	8	8	8	-	RI/MS/O/S
7	*γ*-Terpinene	1240	1238	Woody, lemon-like	-	2	2	4	2	-	RI/MS/O
8	(*E*)-*β*-Ocimene	1248	1250	Sweet, herbal	16	8	8	32	16	8	RI/MS/O/S
9	*β*-Ocimene	1252	1252	Floral	2	2	2	4	4	2	RI/MS/O/S
10	*p*-Cymene	1276	1272	Spicy	-	-	-	4	2	2	RI/MS/O
11	(*E*)-2-Heptenal	1334	1334	Fatty	8	8	8	8	16	16	RI/MS/O/S
12	Nonanal	1387	1396	Fatty	2	2	2	2	2	4	RI/MS/O
13	Acetic acid	1448	1424	Sour	64	64	32	64	64	64	RI/MS/O/S
14	Furfural	1459	1466	Bready	-	2	2	-	-	4	RI/MS/O
15	(*E*,*E*)-2,4-Heptadienal	1502	1497	Fatty	2	-	-	2	2	2	RI/MS/O
16	Benzaldehyde	1520	1527	Sweet	-	2	2	2	2	2	RI/MS/O
17	Linalool	1557	1552	Spicy, citrus-like, woody	128	128	128	8	64	64	RI/MS/O/S
18	Linalyl acetate	1562	1525	Woody, herbal	32	8	128	8	64	128	RI/MS/O/S
19	Caryophyllene	1613	1570	Spicy	16	8	8	-	16	8	RI/MS/O/S
20	Terpinen-4-ol	1638	1635	Spicy, musty	8	16	8	16	8	16	RI/MS/O/S
21	(*E*)-2-Decenal	1640	1636	Cowy	8	8	8	8	8	8	RI/MS/O/S
22	*α*-Terpinyl acetate	1681	1770	Herbal, citrus-like	8	8	8	16	8	8	RI/MS/O/S
23	*α*-Terpineol	1695	1680	Pine-like, woody	-	16	8	16	8	8	RI/MS/O/S
24	Geranyl acetate	1731	1731	Floral	2	2	2	2	-	2	RI/MS/O
25	Piperitone	1756	1743	Herbal, minty	-	-	-	4	4	4	RI/MS/O
26	(*E*,*E*)-2,4-Decadienal	1807	1789	Fatty	16	16	16	16	16	16	RI/MS/O/S
27	Anethole	1821	1817	Minty	2	-	-	-	2	2	RI/MS/O

^a^ Compounds were numbered consecutively based on RI values and their classification as aroma compounds. ^b^ Retention Index of DB-WAX capillary column. ^c^ Theoretical RI as consulted on the NIST Chemistry WebBook. ^d^ Odor perception as perceived by GC-O. ^e^ The “-” indicates that the aroma compound was not smelled or detected. ^f^ The methods of identification compared were mass spectrometry (MS), retention index (RI), olfactory method (O), and standard compound method (S).

**Table 2:** Concentrations and OAVs of key aroma compounds in six varieties of Sichuan pepper tallow hotpots.

Compounds	Linear Equations	R^2^	Concentration (μg/g) ^a^							OAV ^c^					
			HYGSP	JJGSP	JYGSP	HCRSP	HYRSP	WDRSP	Odor Threshold (μg/g) ^b^	HYGSP	JJGSP	JYGSP	HCRSP	HYRSP	WDRSP
Linalool	y = 0.338x + 0.0062	0.992	200.27 ± 2.73	148.58 ± 3.63	240.74 ± 13.47	10.66 ± 0.74	67.72 ± 0.32	52.96 ± 1.85	1.08	185	175	223	10	63	49
Linalyl acetate	y = 0.2232x − 0.0032	0.997	52.45 ± 30.31	13.43 ± 1.18	121.05 ± 10.34	6.32 ± 1.01	86.15 ± 0.42	136.91 ± 1.76	1.13	46	12	107	6	76	121
*D*-Limonene	y = 0.096x − 0.0005	0.996	209.9 ± 10.52	141.64 ± 1.93	268.84 ± 5.58	149.68 ± 8.03	350.67 ± 16.08	58.04 ± 5.8	2.1	100	67	128	71	167	28
*β*-Myrcene	y = 0.1786x − 0.0005	0.998	44.05 ± 0.77	23.68 ± 0.24	57.92 ± 0.93	86.32 ± 6.9	71.42 ± 0.15	16.35 ± 2.39	0.67	66	35	86	129	107	24
Sabinene	y = 0.3683x − 0.0014	0.999	31.88 ± 0.43	5.88 ± 0.05	21.12 ± 0.73	7.11 ± 0.51	2.64 ± 0.08	3.39 ± 0.51	0.98	33	6	22	7	3	3
Eucalyptol	y = 0.2041x − 0.0019	0.999	-	-	-	18.48 ± 1.88	8.98 ± 1.34	3.97 ± 0.34	1.15	-	-	-	16	8	3
*α*-Terpineol	y = 0.2103x − 0.0008	0.999	-	5.2 ± 0.11	3.31 ± 0.04	8.37 ± 0.32	3.02 ± 0.84	5.52 ± 0.48	0.47	-	11	7	18	6	12
Terpinen-4-ol	y = 0.2167x + 0.0008	0.997	2.93 ± 0.05	4.76 ± 0.14	4.49 ± 0.11	28.77 ± 0.73	0.95 ± 0.17	7.38 ± 0.63	0.29	10	16	15	99	3	25
Acetic acid	y = 0.1945x − 0.0041	0.999	7.35 ± 1.18	8.48 ± 0.66	5.03 ± 0.02	7.34 ± 0.21	8.77 ± 0.1	7.93 ± 0.22	0.13	57	65	39	56	67	61
(*E*)-*β*-Ocimene	y = 0.5782x − 0.0634	0.998	2.15 ± 0.02	0.22 ± 0.01	0.26 ± 0.01	6.01 ± 0.6	1.61 ± 0.04	0.53 ± 0.03	0.034	63	6	8	177	47	15
(*E*,*E*)-2,4-Decadienal	y = 0.7926x − 0.1262	0.997	6.26 ± 0.37	5.72 ± 0.71	6.51 ± 0.29	5.46 ± 0.43	5.19 ± 0.53	6.18 ± 0.77	2.15	3	3	3	3	2	3
*β*-Phellandrene	y = 0.1725x + 1.7557	0.999	4.38 ± 0.18	2.46 ± 0.12	5.17 ± 0.11	13.48 ± 0.67	1.09 ± 0.08	0.09 ± 0.03	0.036	122	68	143	374	30	2
*β*-Caryophyllene	y = 0.6714x + 0.0068	0.991	1.09 ± 0.01	0.52 ± 0.03	0.71 ± 0.04	−	1.3 ± 0.04	0.84 ± 0.04	0.064	17	8	11	−	20	13
*α*-Terpinyl acetate	y = 0.265x + 0.0258	0.996	2.95 ± 0.28	3.03 ± 0.23	5.13 ± 0.51	10.91 ± 0.19	1.87 ± 0.01	1.6 ± 0.06	0.48	6	6	11	23	4	3
(*E*)-2-Heptenal	y = 0.7547x + 0.0181 R = 0.994	0.994	1.22 ± 0.03	1.16 ± 0.04	1.24 ± 0.03	1.25 ± 0.05	2.01 ± 0.04	1.76 ± 0.08	0.001	1222	1163	1238	1250	2015	1757
(*E*)-2-Decenal	y = 0.6213x + 0.1044	0.998	1.09 ± 0.01	0.74 ± 0.04	0.75 ± 0.05	0.72 ± 0.05	1.03 ± 0.08	0.9 ± 0.04	0.39	3	2	2	2	3	2

^a^ The “-” indicates that the concentration of the aroma compounds was not detected. ^b^ The odor threshold was determined based on the literature concerning the odor threshold of odorants in oil [14,16,21]. ^c^ The “-” indicates that the OAV of the aroma compounds was not detected.

## Data Availability

The original contributions presented in this study are included in the article/Supplementary Materials. Further inquiries can be directed to the corresponding authors.

## Supplementary Materials

The following supporting information can be downloaded at: https://www.mdpi.com/article/10.3390/foods14040627/s1, Figure S1. Six cultivars of Sichuan pepper; Table S1. The main aroma compounds of Sichuan pepper pericarp oil, raw Sichuan pepper, and stir-fried Sichuan pepper tallow; Table S2. Volatile compounds in beef tallow Relative Proportions.
