# Supplementary material for: Characterization of Volatile Compounds and Odorants in Different Sichuan Pepper Varieties in Tallow Hotpot
Source: Foods. 2025 Feb 13;14(4):627. doi: 10.3390/foods14040627 (PMC11854310; doi:10.3390/foods14040627)
Supplement: Supplementary file 1 [file foods-14-00627-s001.zip › foods-3453159-supplementary.pdf]

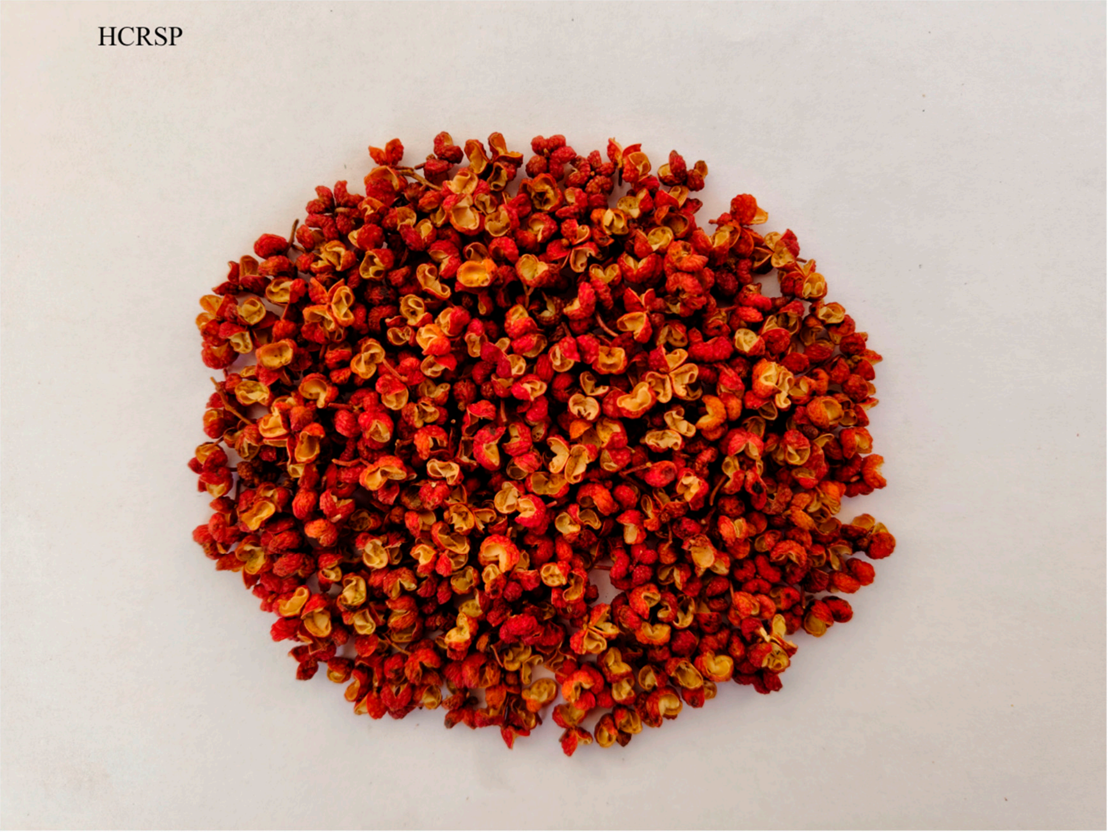
HCRSP

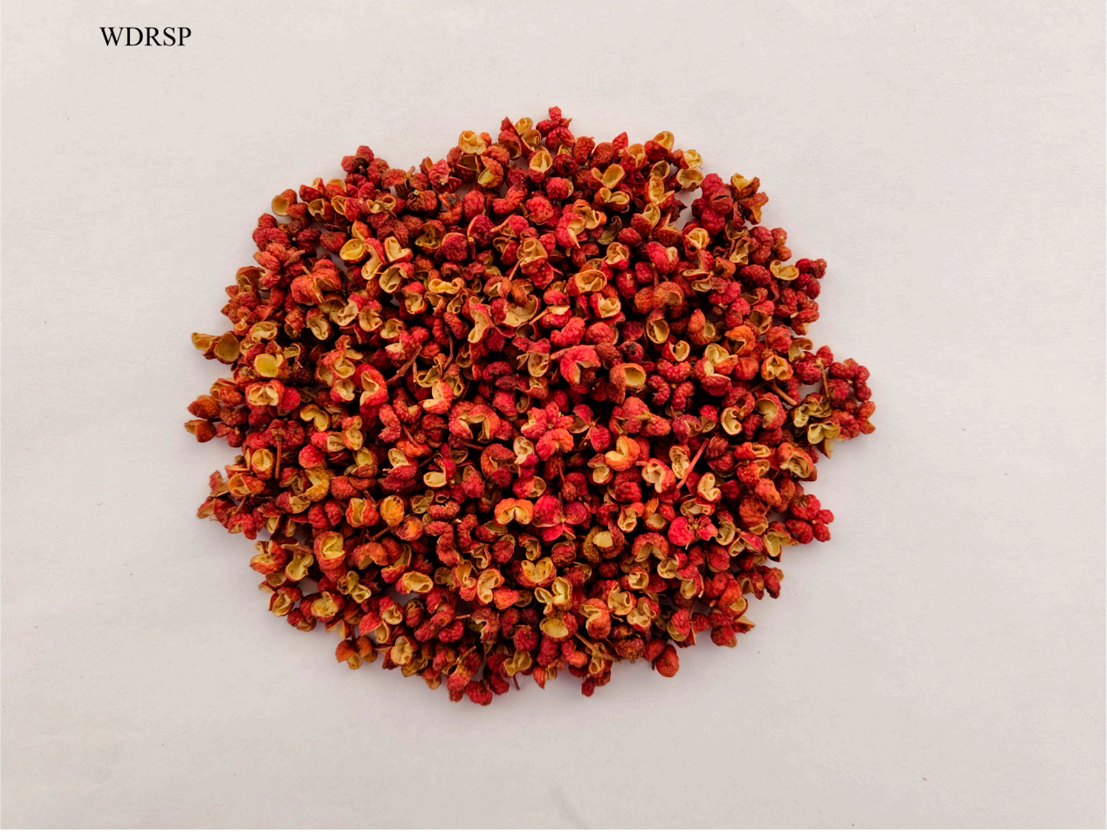
WDRSP

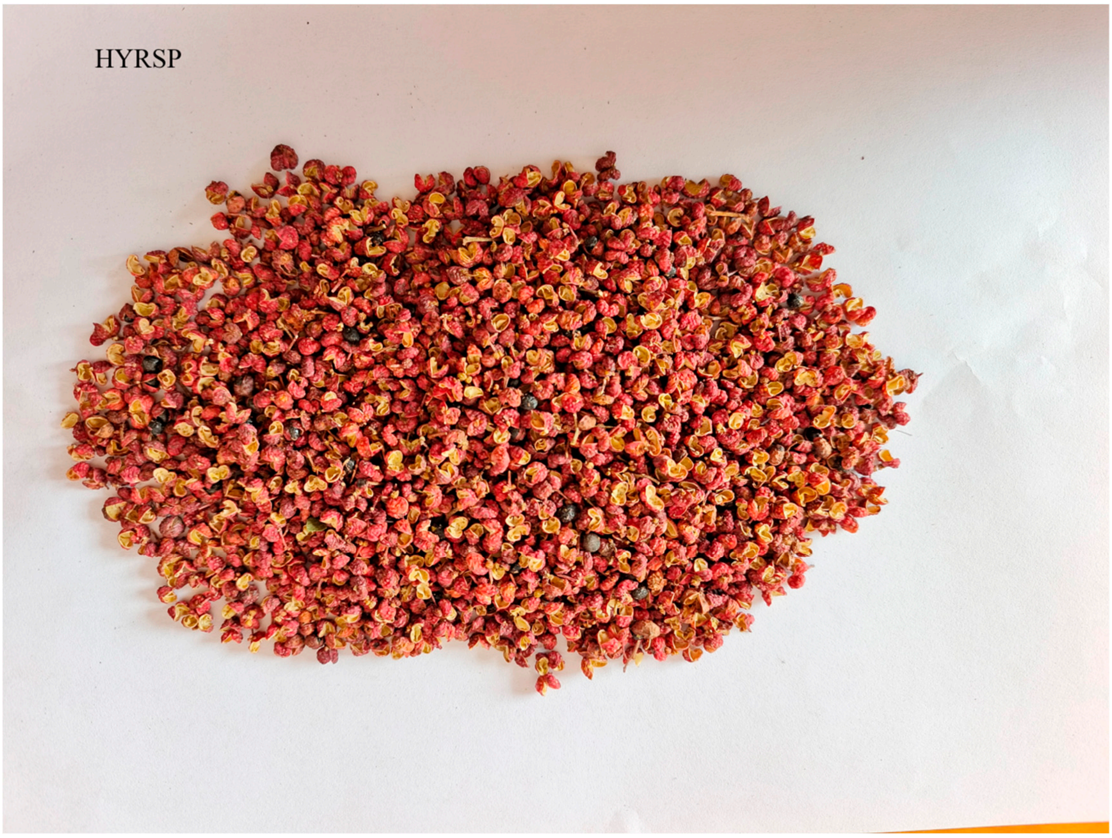
HYRSP

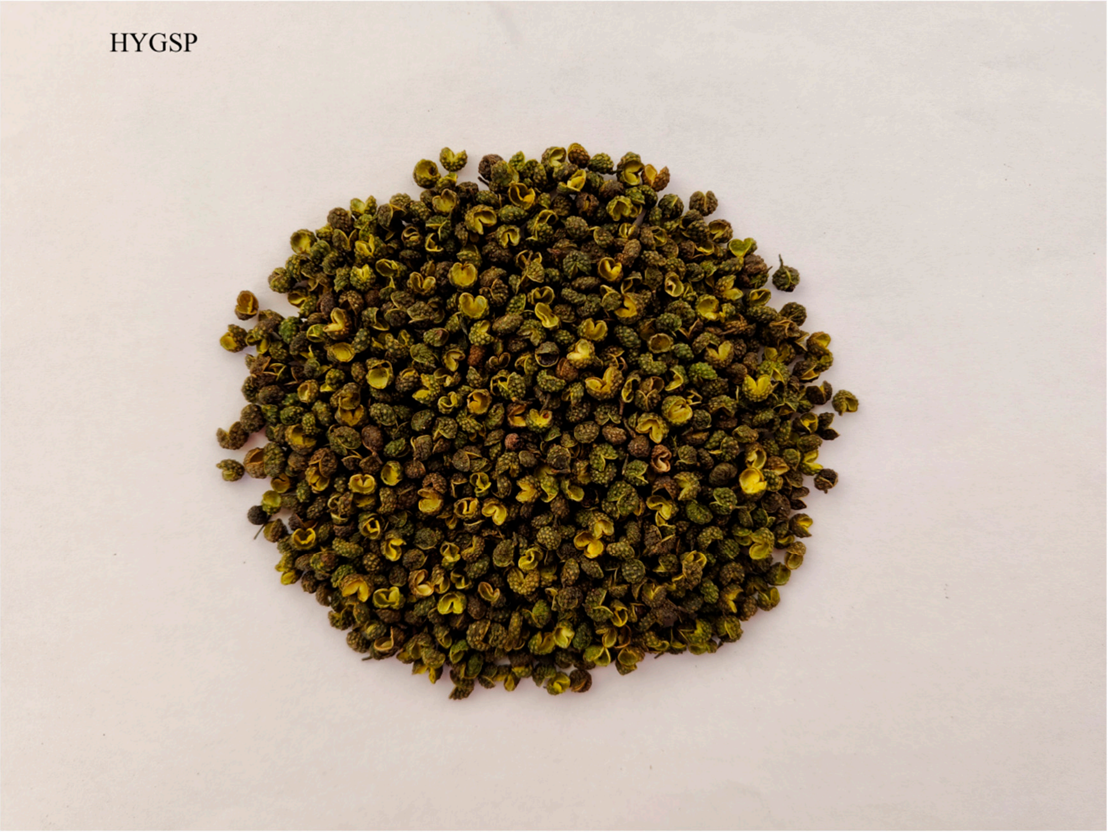
HYGSP

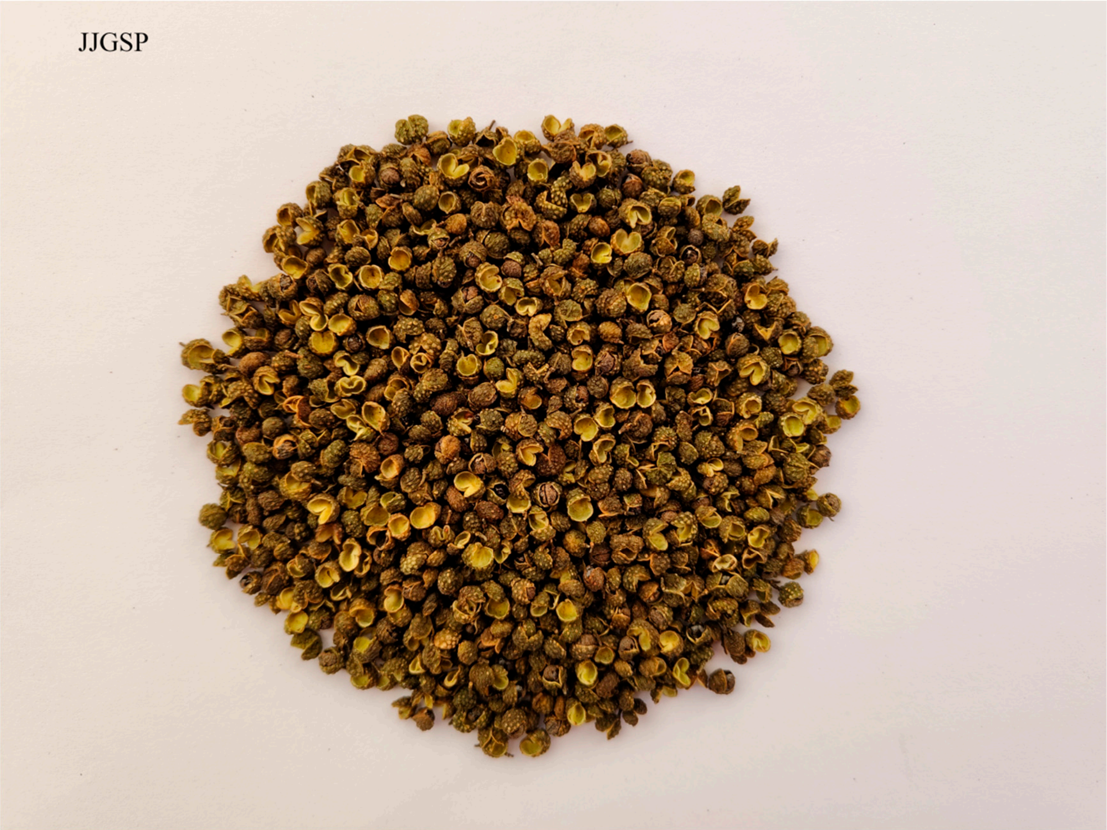

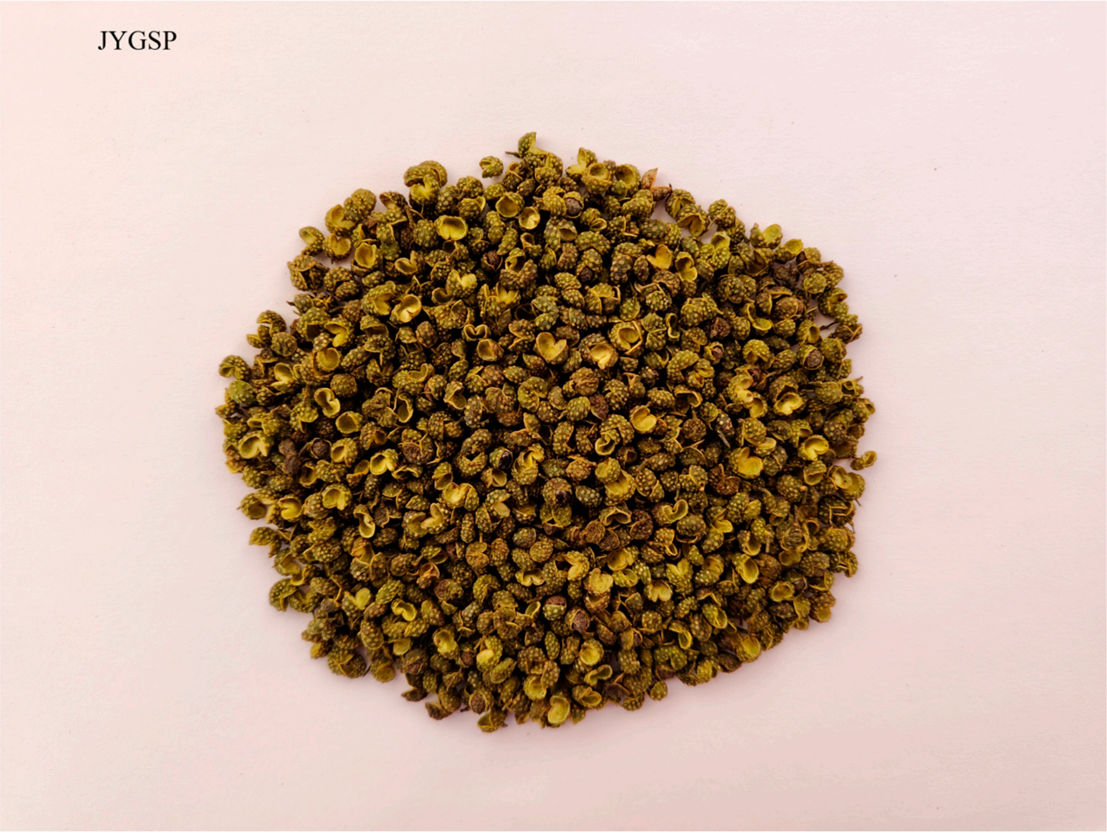

Figure S1. Six cultivars of Sichuan pepper.

Hanyuan green Sichuan pepper (HYGSP), Jiangjin green Sichuan pepper (JJGSP), Jinyang green Sichuan pepper (JYGSP), Hancheng red Sichuan pepper (HCRSP), Wudu red Sichuan pepper (WDRSP), Hanyuan red Sichuan pepper (HYRSP).

Table S1. The main aroma compounds of Sichuan pepper pericarp oil, raw Sichuan pepper, and stir-fried Sichuan pepper tallow.

Compounds	Concentration (μg/g)[a]		
	**Sichuan pepper pericarp oil**	**Raw Sichuan pepper**	**Stir-fried Sichuan pepper tallow**
**Terpenes**			
(+)-Limonene/D-Limonene	42.53 ± 0.09	2809.1 ± 222.00	81.52 ± 10.95
α-Terpinene	3.24 ± 0.03	25.2 ± 2.00	-
γ-Terpinene	1.60 ± 0.01	71.4 ± 6.00	3.50 ± 0.30
(*E*)-*β*-Ocimene	0.42 ± 0.00	875.80 ± 64.80	-
β-Myrcene	0.43 ± 0.00	1366.80 ± 106.30	-
Allo-ocimene	0.63 ± 0.00	-	-
β-Pinene	0.13 ± 0.00	38.80 ± 3.30	-
β-Caryophyllene	0.44 ± 0.01	116.60 ± 11.10	-
Terpinolene	0.38 ± 0.01	-	-
α-Phellandrene	0.07 ± 0.00	176.50 ± 12.80	-
α-Thujene/(–)-thujone	0.14 ± 0.00	-	-
γ-Muurolene	0.03 ± 0.00	-	-
(E)-Sabinene	-	-	35.71 ± 7.57
**Alcohols**			
Linalool	12.98 ± 0.11	211.30 ± 14.60	872.80 ± 14.64
α-Terpineol	-	-	3.12 ± 0.27
Eucalyptol	-	-	15.86 ± 3.76
Terpinen-4-ol	0.37 ± 0.00	-	42.21 ± 1.01
Phenylethyl alcohol	0.03 ± 0.00	-	4.24 ± 1.14
(*E*)-4-Thujanol	-	-	13.08 ± 0.63
Geraniol	0.11 ± 0.00	-	-
**Esters**			
Linalyl acetate	-	531.90 ± 42.20	284.43 ± 9.67
α-Terpinyl acetate	-	-	14.70 ± 0.99
Phenylethyl acetate	-	-	0.56 ± 0.09
Geranyl acetate	0.01 ± 0.00	-	-
Cuminaldehyde	0.01 ± 0.00	-	-
2-Phenethyl acetate	0.03 ± 0.00	-	-
Methyl vanillate	-	-	1.25 ± 0.01
**Aldehydes**			
(*E*)-Cinnamaldehyde	-	-	5.45 ± 1.26
Decanal	0.02 ± 0.00	-	-
(*E,E*)-2,4-Hexadienal	-	-	0.03 ± 0.00
(*E*)-2-Decenal	-	-	0.35 ± 0.30
(*E,E*)-2,4-Decadienal	-	-	5.45 ± 0.32
Undecanal	0.38 ± 0.00	-	-
Citronellal	91.93 ± 0.06	-	-
**others**			
D-Carvone	-	-	5.72 ± 0.32
Acetic acid	-	-	13.75 ± 1.15
(E)-Anethol	-	-	20.99 ± 5.70

[a] The "-" indicates that the concentration of the aroma compounds were not detected. The table data is sourced from literature [1,2,3].

Table S2. Volatile compounds in beef tallow Relative Proportions

Compounds	Relative content (%)
Hexanal	18.16
Octanal	19.87
Nonanal	34.11
(*E*)-2-Nonenal	7.89
(*E*)-2-Decenal	5.06
(*E*)-2-Undecenal	5.99
(*E,E*)-2,4-Decadienal	2.32
2-Pentylfuran	6.60

References

1. Liu, Y.P.; Li, Q.R.; Yang, W.X.; Sun, B.G.; Zhou, Y.; Zheng, Y.; Huang, M.Q.; Yang, W.J. Characterization of the potent odorants in *Zanthoxylum armatum* DC Prodr. pericarp oil by application of gas chromatography-mass spectrometry-olfactometry and odor activity value. *Food Chem*. **2020**, *319*, 8.
2. Shao, Y.Y.; Liu, X.Q.; Zhang, Z.Y.; Wang, P.X.; Li, K.K.; Li, C.M. Comparison and discrimination of the terpenoids in 48 species of huajiao according to variety and geographical origin by E-nose coupled with HS-SPME-GC-MS. *Food Research International* **2023**, *167*, 12.
3. Zhang, W.B.; Chen, C.; Li, Y.X.; Guo, F.Y.; Liu, W.Q.; Liu, S.Y.; Sun, Y.N.; Wang, X.F.; Shen, Y.M.; Wang, P.J. Analysis of composition and source of the key aroma compounds in stir-fried pepper tallow. *Food Chem*. **2024**, *441*, 7.
